# Diffusion tensor imaging and arterial tissue: establishing the influence of arterial tissue microstructure on fractional anisotropy, mean diffusivity and tractography

**DOI:** 10.1038/s41598-020-77675-x

**Published:** 2020-11-26

**Authors:** B. Tornifoglio, A. J. Stone, R. D. Johnston, S. S. Shahid, C. Kerskens, C. Lally

**Affiliations:** 1 Trinity Centre for Biomedical Engineering, Trinity Biomedical Sciences Institute, Trinity College Dublin, Dublin, Ireland; 2 Department of Mechanical, Manufacturing and Biomedical Engineering, School of Engineering, Trinity College Dublin, Dublin, Ireland; 3 Department of Radiology and Imaging Sciences, Indiana University School of Medicine, Indianapolis, IN USA; 4 Trinity College Institute of Neuroscience, Trinity College Dublin, Dublin, Ireland; 5 Advanced Materials and Bioengineering Research Centre (AMBER), Royal College of Surgeons in Ireland and Trinity College Dublin, Dublin, Ireland

**Keywords:** Biophysics, Biomarkers, Cardiology, Diseases, Engineering, Materials science

## Abstract

This study investigates diffusion tensor imaging (DTI) for providing microstructural insight into changes in arterial tissue by exploring how cell, collagen and elastin content effect fractional anisotropy (FA), mean diffusivity (MD) and tractography. Five ex vivo porcine carotid artery models (n = 6 each) were compared—native, fixed native, collagen degraded, elastin degraded and decellularised. Vessels were imaged at 7 T using a DTI protocol with b = 0 and 800 s/mm^2^ and 10 isotopically distributed directions. FA and MD were evaluated in the vessel media and compared across models. FA values measured in native (p < 0.0001), fixed native (p < 0.0001) and collagen degraded (p = 0.0018, p = 0.0016, respectively) were significantly higher than those in elastin degraded and decellularised arteries. Native and fixed native had significantly lower MD values than elastin degraded (p < 0.0001) and decellularised tissue (p = 0.0032, p = 0.0003, respectively). Significantly lower MD was measured in collagen degraded compared with the elastin degraded model (p = 0.0001). Tractography yielded helically arranged tracts for native and collagen degraded vessels only. FA, MD and tractography were found to be highly sensitive to changes in the microstructural composition of arterial tissue, specifically pointing to cell, not collagen, content as the dominant source of the measured anisotropy in the vessel wall.

## Introduction

Stroke and ischaemic heart disease are the most prevalent forms of cardiovascular disease^1^, while atherosclerosis is widely accepted as the most significant contributor to these burdens^2^. Although numerous mechanisms are associated with the progression of atherosclerosis, changes in vessel microstructure are implicated at the early stages of disease onset^3,4^. As such, imaging markers that are sensitive to changes in arterial tissue microstructure have the potential to provide unique insight into disease onset and progression.

Diffusion tensor imaging (DTI) offers a non-invasive method to probe tissue microstructure, providing quantitative metrics such as mean diffusivity (MD) and fractional anisotropy (FA) which describe the interaction between proton diffusion and the underlying tissue structure. While DTI has predominantly been used to examine white matter, its application in tissue outside the brain has taken significant strides in recent years. To date, a handful of studies have explored the application of DTI to arterial tissue^5,6^ and demonstrated its sensitivity to changes in tissue integrity^7–9^. These studies have laid the groundwork and demonstrated both the feasibility and promise of DTI to effectively investigate underlying tissue microstructure in arterial vessels. However, the effect that microstructural changes have on key diffusion metrics remains unanswered. In a multifaceted microstructure like that of arterial tissue, understanding the impact of the different tissue constituents is critical to inferring any clinically relevant insights from DTI measurements.

Healthy arterial tissue is composed of three main layers: the intimal, medial and adventitial layers. Regardless of location in the body, these layers are predominantly composed of smooth muscle cells (SMCs), collagen and elastin in varying degrees^10^. SMCs attach to elastin fibres and arrange obliquely between concentric lamellae^11^. These SMCs are responsible for the turnover of collagen in the extracellular matrix. This collagen—predominantly type I—is the main load-bearing component of arterial tissue^12^. The close relationship between elastin, SMCs and collagen, together form a continuous helically arranged matrix^10,11^, whose ability to withstand forces both circumferentially and axially allow for the proper mechanical function of healthy arterial tissue. The quantity, quality and arrangement of these components can be disrupted during disease progression and result in significant mechanical shortcomings and failings^4,13,14^.

Fibre tractography has previously been reported to show the helical arrangement of healthy arterial tissue microstructure^5,6^, as well as the disruption to these highly organised tracts when the underlying microstructure is damaged^7^ and the high variability of fibre arrangements in an atherosclerotic plaque^15^. Additionally, Shahid et al.^8^ reported decreased FA values when altering healthy arterial tissue by cutting it and maintaining it open. However, the specificity of FA, MD and tractography to individual microstructural components, such as SMCs, collagen and elastin, remains unclear in arterial tissue, therefore, impeding the interpretation of such metrics.

Previous studies on articular cartilage have looked at FA, MD and fibre tractography through the thickness of cartilage^16^ and between anterior and posterior ligaments^17^, where the degree of collagen alignment differs, as well as in damaged cartilage^18,19^, where collagen orientation is disrupted. While these studies look at the global influence of changing tissue structure on DTI metrics, they all show changes in FA, MD and tractography which correlate well, morphologically, to changes in collagen content and arrangement. The sensitivity of DTI metrics to changes in microstructure from degradation treatments in articular cartilage, which is rich in collagen and proteoglycans but has low cell content^20^, highlight the potential insight that a more selective, component-specific treatment could yield^21^. Fibre tractography has also been shown to be sensitive to the time-dependent orientation of collagen fibres in biodegradable tissue engineered constructs seeded with human-derived vascular cells^22^. Similarly, in cardiac tissue which has a laminar architecture composed of myofibers and collagen fibres, the orientation of cardiomyocytes^23,24^ has been illustrated by tractography, as well as the differentiation between healthy and diseased cardiac architecture^25–27^. Together, these studies allude to the ability of DTI metrics to be selectively sensitive to specific microstructural components, but this has yet to be conclusively determined in arterial tissue.

The aim of this study is to investigate the potential of DTI to provide specific insight into microstructural changes in arterial tissue by exploring the influence of key components on FA, MD and fibre tractography. This is achieved using ex vivo porcine carotid artery (PCaA) models, developed to selectively remove individual elements of arterial microstructure—SMCs, elastin and collagen. Comparing FA, MD and tractography across these models allows for microstructural insight using DTI metrics. These metrics have the potential to yield novel characterisation of both arterial health and disease progression.

## Results

### Tissue model validation

**Figure 1:**
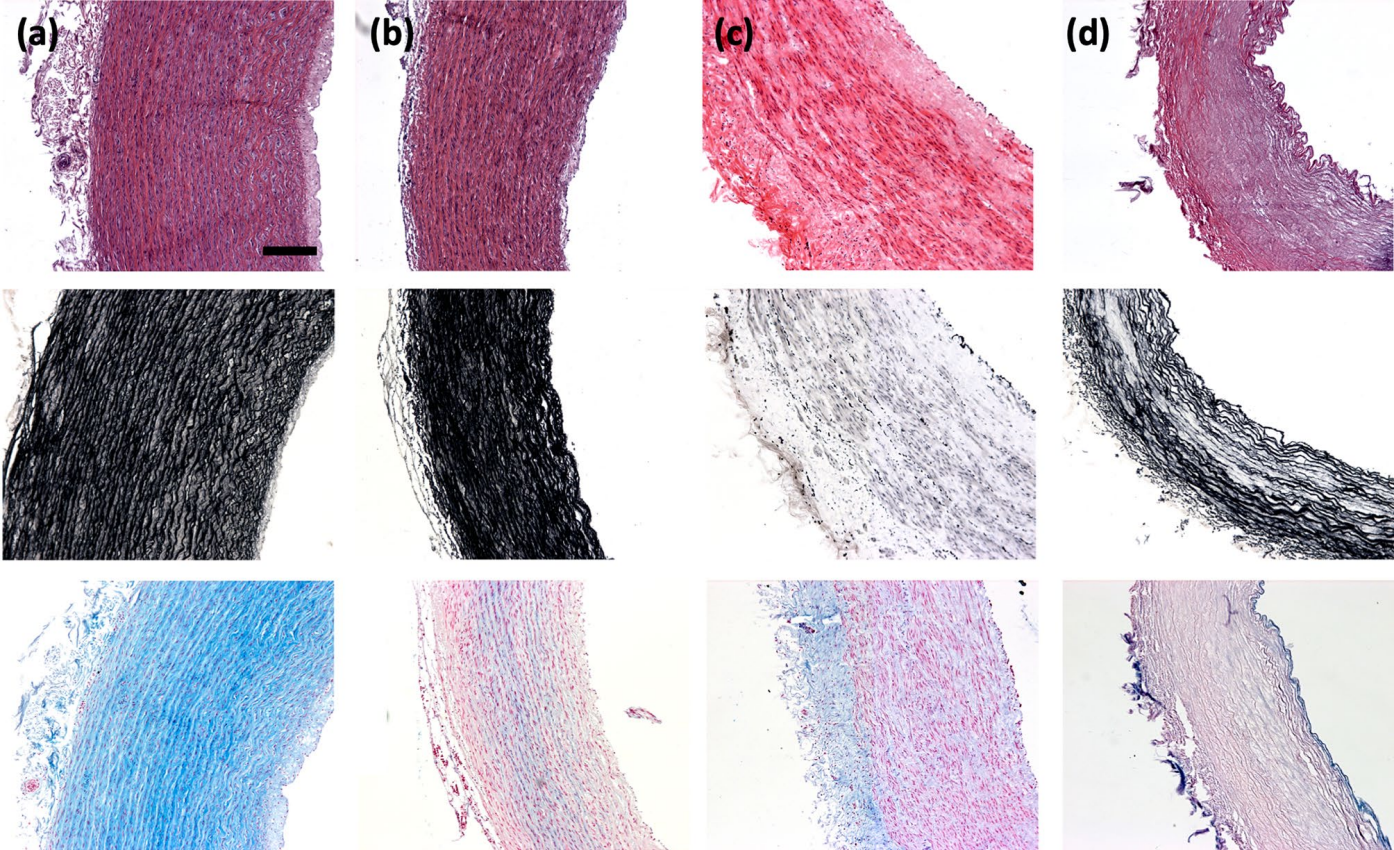
Histological representations of tissue models. Representative cell, elastin and glycosaminoglycans in (**a**) native, (**b**) collagen degraded, (**c**) elastin degraded and (**d**) decellularised PCaA. Top to bottom: cell content visible by purple-blue nuclei in H&E, elastin shown in Verhoeff’s elastin stain in black and GAGs stained blue by Alcian blue. All imaged using brightfield microscopy. Scale bar 250 μm.

Five tissue models were used in this study to investigate the sensitivity of DTI to the microstructural components of arterial tissue. The fixed native PCaA model is not presented in the histological figures as all PCaA models are fixed prior to histological processing, making it redundant compared to histology of native PCaA. Figure 1 shows Haematoxylin and Eosin (H&E), Verhoeff’s and Alcian blue staining for the tissue models. In order to truly understand the influence of each of the tissue components, the selective removal of individual microstructural components was necessary and is confirmed here. H&E verifies cellular content remained in all model tissues, with the exception of decellularised (Fig. 1d, top row)—where the complete removal of cells is confirmed. Similarly, the Verhoeff’s elastin stain validates that elastin was removed in the elastin degraded model only (Fig. 1c, middle row). Alcian blue (Fig. 1, bottom row) shows a variety of glycosaminoglycan (GAG) concentrations throughout the models. While this hasn’t been investigated in arterial tissue, GAGs have been shown to leach out of tissue when immersed in phosphate buffered saline (PBS) in order to establish homeostasis^28^.

**Figure 2:**
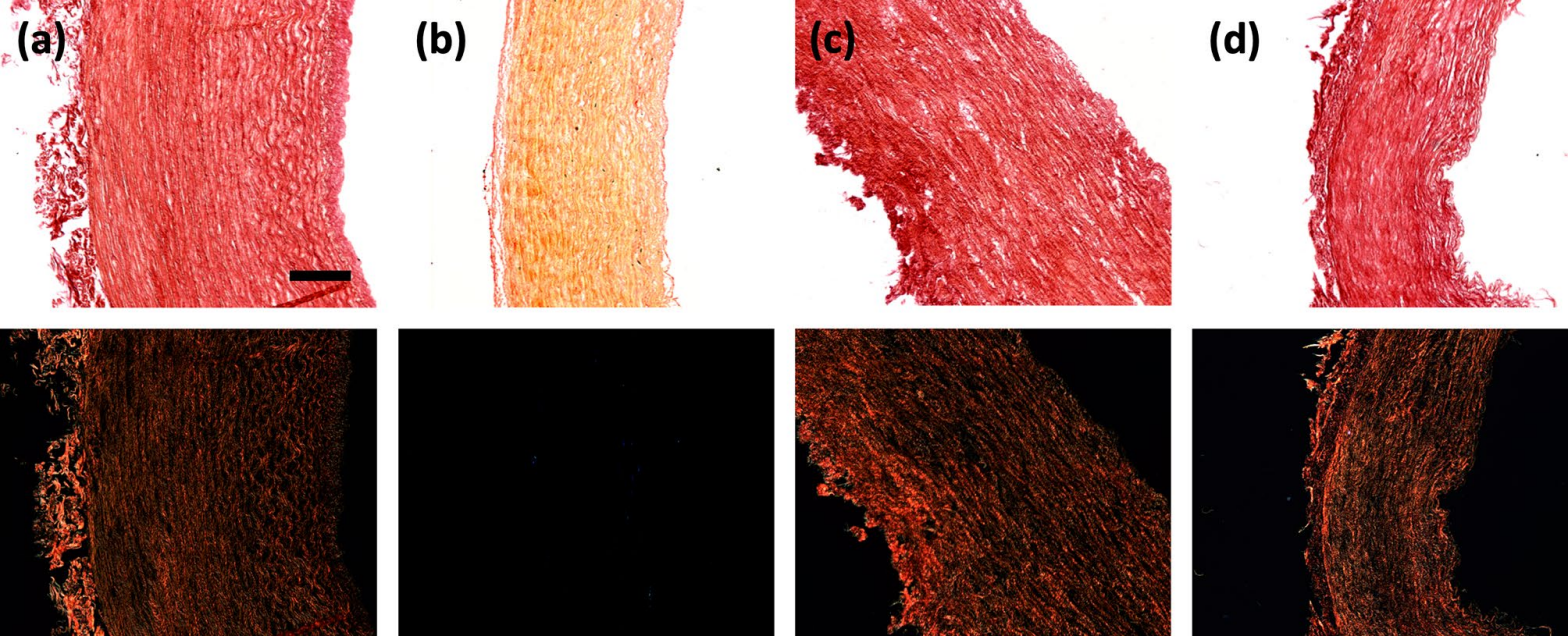
Histological representation of collagen content and orientation. Collagen in (**a**) native, (**b**) collagen degraded, (**c**) elastin degraded and (**d**) decellularised PCaA. Brightfield microscopy (top row) shows all tissue stained red, where the PLM on the bottom row has a specificity for the birefringence of collagen. Scale bar 250 μm.

Figure 2 similarly validates the tissue models, but with respect to collagen content by picrosirius red staining. The top row shows brightfield imaging of the models where collagen is visualised in red. Polarised light microscopy (PLM), in the second row, has a specificity for the birefringence of collagen fibres and therefore gives a representation of collagen fibre orientations. Together these confirm that the collagen degraded model (Fig. 2b) removed all collagen content. These also confirm that while collagen content was not affected in the other models, neither was the collagen orientation.

**Figure 3:**
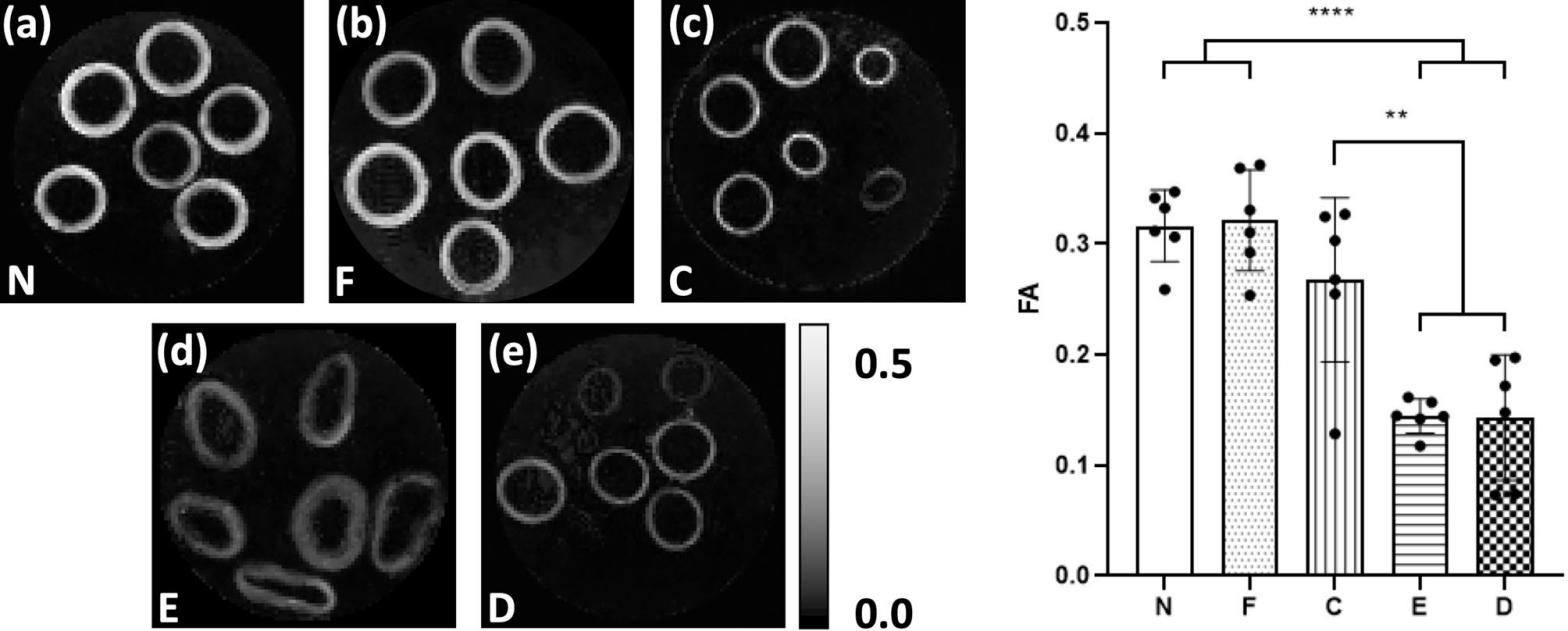
Parametric maps of FA in a representative slice for each of the tissue models. As measured in vessel media, both (**a**) native (N) and (**b**) fixed native (F) PCaA showed significantly higher FA than both the (**d**) elastin degraded (E) and (**e**) decellularised (D) tissue models. (**c**) Collagen degraded PCaA also showed a significantly higher FA than both elastin degraded and decellularised PCaA. FA maps scaled to show 0 to 0.5 (**p = 0.0018 (C vs. E), **p = 0.0016 (C vs. D), ****p < 0.0001).

The mean FA measured in the media of each vessel is grouped by tissue model and presented in Fig. 3 alongside parametric maps of FA in a representative slice for each model. Visually, the FA maps show stark differences between select tissue models. Native, fixed native and collagen degraded PCaA (Fig. 3a–c) appear similar. The elastin degraded PCaA (Fig. 3d) shows the expansion of the vessels and the apparent loss of tightly bound structure seen in native PCaA—this increase in extracellular space can be seen histologically in Fig. 1c. In contrast, both the elastin degraded and decellularised PCaA (Fig. 3e) show lower FA ranges. These observations were confirmed by the mean FA measured in the media of each vessel. Native and fixed PCaA demonstrated significantly higher FA than both the elastin degraded and decellularised PCaA (****p < 0.0001). Additionally, the collagen degraded PCaA maintained a significantly higher FA than elastin degraded (**p = 0.0018) and decellularised (**p = 0.0016). No significant differences were seen between native, fixed or collagen degraded PCaA.

**Figure 4:**
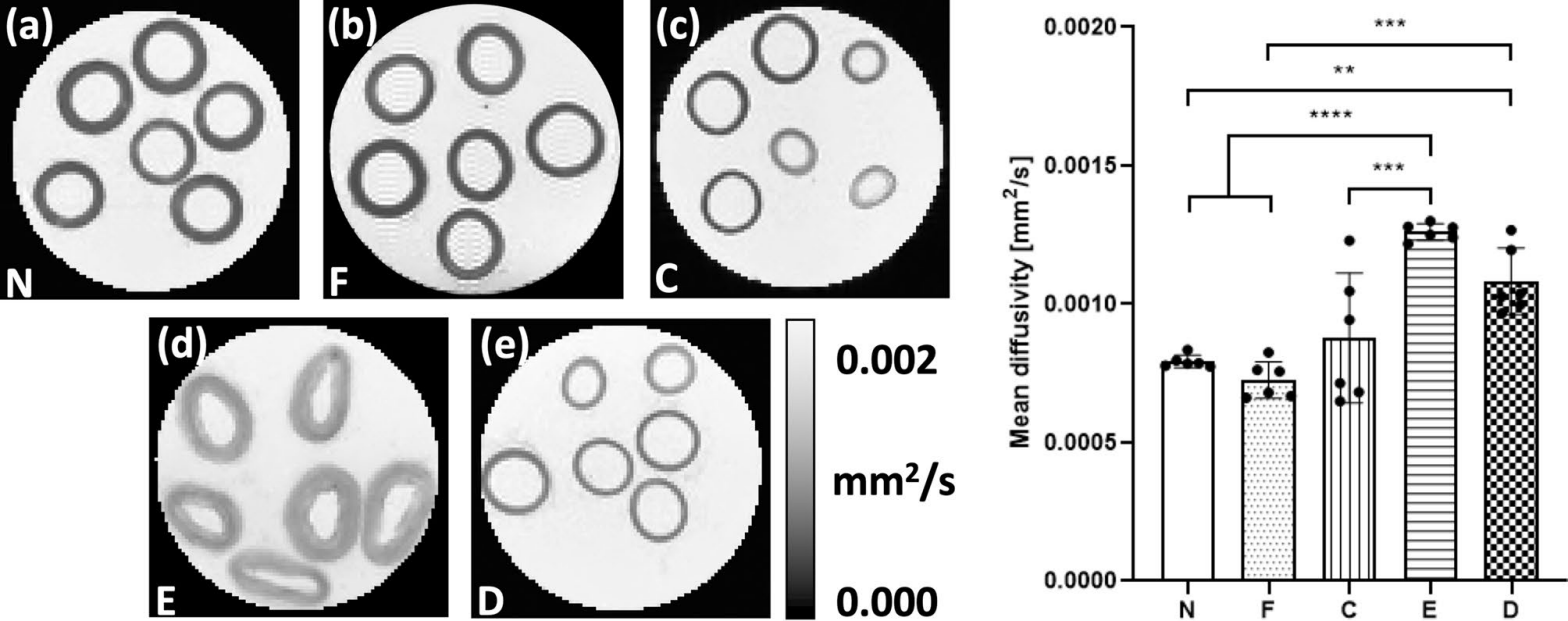
Parametric maps of MD in a representative slice for each of the tissue models. As measured in vessel media, both (**a**) native (N) and (**b**) fixed native (F) PCaA showed a significantly lower MD than both the (**d**) elastin degraded (E) and (**e**) decellularised (D) PCaA. The elastin degraded PCaA had a significantly higher MD than the (**c**) collagen degraded PCaA (**p = 0.0032, ***p = 0.0001 (C vs. E), ***p = 0.0003 (F vs. D), ****p < 0.0001).

Parametric maps of MD and regional values of MD extracted from the vessel media for each tissue model are presented in Fig. 4. Native and fixed PCaA showed significantly lower MD values than the elastin degraded model (****p < 0.0001), which can be seen visually in the MD maps (Fig. 4a,b). Decellularised PCaA (Fig. 4e) had a significantly higher overall diffusion than both native (**p = 0.0032) and fixed tissue (***p = 0.0003). Fixed PCaA had a lower mean MD than native PCaA, however no significant difference was found. The collagen degraded PCaA showed a significantly lower MD than the elastin degraded model (***p = 0.0001), which is evidenced in the MD maps as well (Fig. 4c,d). As the samples were imaged at room temperature, the MD for PBS was found to be 0.00185 ± 0.00001 mm^2^/s.

**Figure 5:**
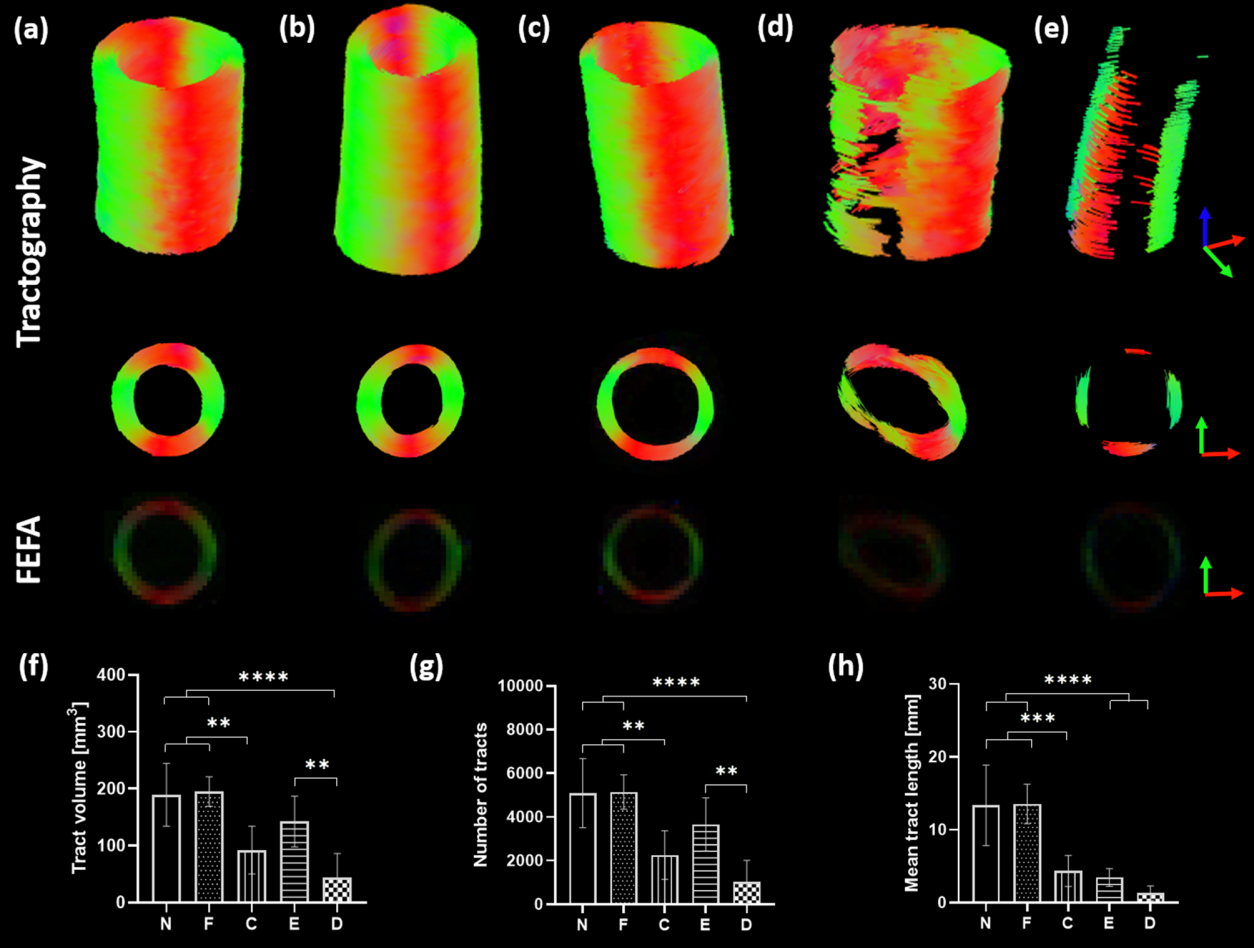
Tractography from tissue models. Tracts from (**a**) native, (**b**) fixed native, (**c**) collagen degraded, (**d**) elastin degraded and (**e**) decellularised PCaA alongside the FEFA maps. All tissue model tractographies were modelled with the following parameters: seed point resolution: 0.3125 mm × 0.3125 mm × 0.3125 mm, FA threshold: 0.1, FA tracking threshold: 0.1–1, tract length: 0.5–50 mm, angular threshold: 30° and step size of 0.3125 mm. Fresh and fixed native PCaA show the most continuous tractography which was verified by (**f**) tract volume (**p = 0.0051 (N vs. C), **p = 0.0029 (F vs. C), **p = 0.0046 (E vs. D), ****p < 0.0001) (**g**) the number of tracts (**p = 0.0025 (N vs. C), **p = 0.0021 (F vs. C), **p = 0.0054 (E vs. D), ****p < 0.0001) and (**h**) and the mean tract length (***p = 0.0002), ****p < 0.0001).

Tractography was performed to visualise the diffusion pathways within the tissue models. Keeping tractography parameters constant, Fig. 5 demonstrates the varying results obtained across tissue models alongside the first eigenvector-fractional anisotropy (FEFA) maps. Fresh and fixed native and collagen degraded PCaA (Fig. 5a–c) illustrate coherent and helical arrangements of tracts which align with the known helical arterial tissue structure. Using the same parameters, the elastin degraded and decellularised PCaA models (Fig. 5d,e) show fewer tracts and lack continuity. Tract volume, the number of tracts and mean tract length were quantified and both fresh and fixed native showed the highest volume and number of tracts as well as the longest tracts (Fig. 5f–h).

**Figure 6:**
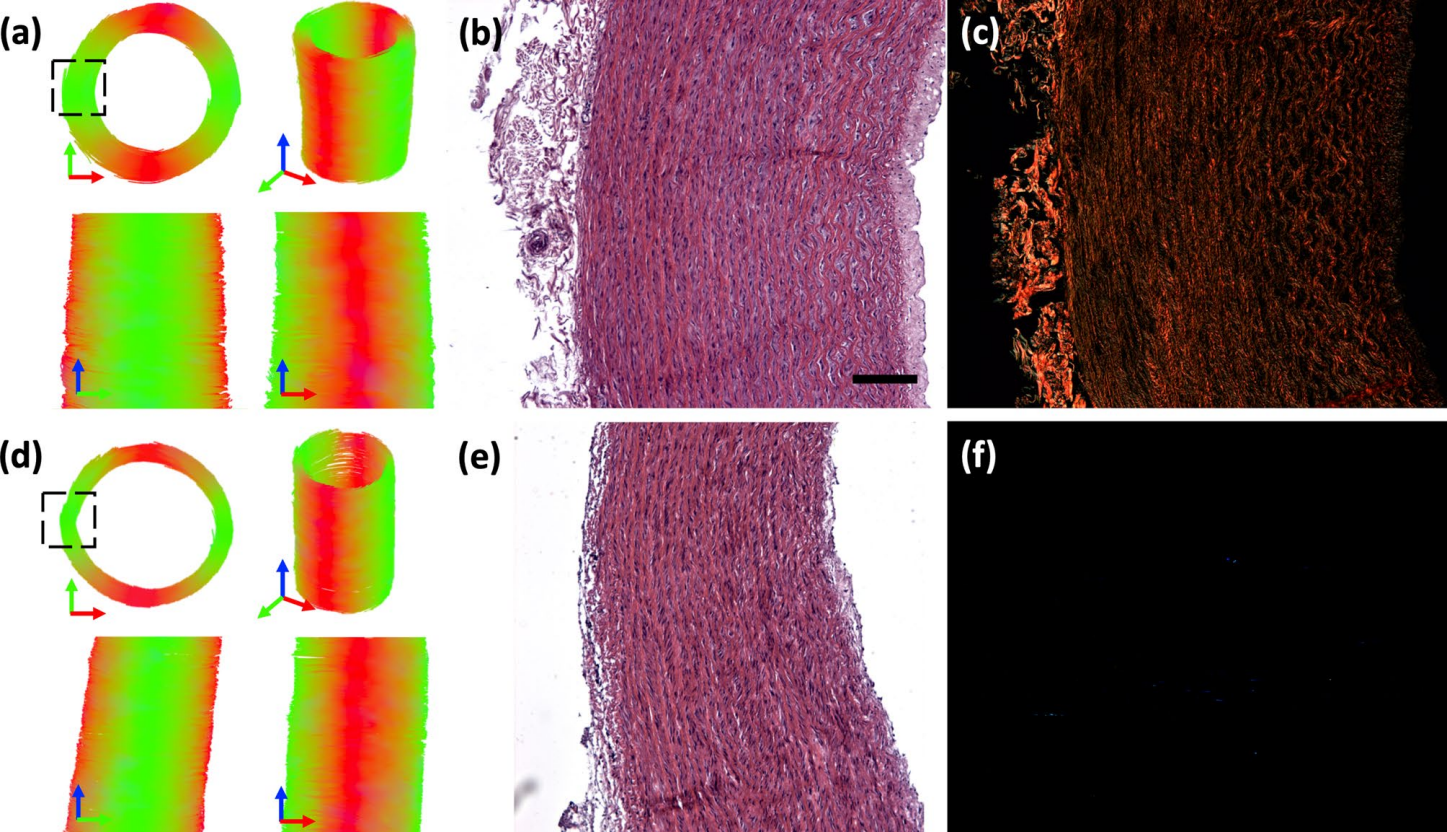
Tractography and histology representations for native and collagen degraded vessels. Tractography of representative (**a**) native and (**d**) collagen degraded PCaA. Both models were obtained with the following parameters: seed point resolution: 0.3125 mm × 0.3125 mm × 0.3125 mm, FA threshold: 0.2, FA tracking threshold: 0.2–1, tract length: 0.5–5.0 mm, angular threshold: 30° and step size of 0.3125 mm. Representative cross-sectional histology shows (**b**,**e**) cellular arrangement by H&E for native and collagen degraded PCaA, respectively. (**c**) PLM shows the similar orientation of collagen in native PCaA, and lack thereof in (**f**) collagen degraded PCaA. Scale bar is 250 μm.

Comparing native and collagen degraded PCaA models, Fig. 6 shows tractography for a representative vessel of each model alongside H&E and PLM histology. A cross-sectional view of native PCaA shows the circumferentially aligned cell and collagen fibre content (Fig. 6b,c). The histologically verified orientation of both cells and collagen coincide well with the arrangement of the tracts of native PCaA (Fig. 6a). The collagen degraded PCaA resulted in similar tract orientation (Fig. 6d), despite the lack of any collagen (Fig. 6f), while the circumferentially aligned cell content is still visible (Fig. 6e).

## Discussion

In the present study we investigated the sensitivity of DTI derived FA, MD and tractography to changes in arterial tissue microstructure. By selectively removing SMCs, elastin and collagen we explored how each component plays a part in the typically anisotropic diffusion profile of arterial tissue^8^ (Fig. 3). Differences between native and decellularised arterial tissue demonstrate that the main contributor to this anisotropic diffusion in arterial tissue is the presence of cell content. While the elastin degraded model suggests a similar contribution from elastin—the removal of elastin resulted in a significant increase in extracellular space and decrease in SMC density. The degree of extracellular space increase in the elastin degraded model was far greater than any other tissue model, making it difficult to elucidate the exact impact elastin has on the anisotropic diffusion of arterial tissue. While previous studies highlight the role of collagen fibres in diffusion derived metrics^8,22,29^, here, we evidence the co-dependency of collagen and cell content and characterise their influence on FA, MD and tractography. With the removal of collagen there is no significant change in FA or MD compared to native arterial tissue. However, the loss of cellular content results in predominantly more isotropic diffusion, seen by increased MD and decreased FA, despite the presence of collagen fibres as confirmed by histology. This becomes even more evident in the tractography results—where the decellularised vessels yield few helically arranged tracts (Fig. 5e–h). Fresh and fixed native vessels resulted in the most uniform, continuously helical tracts (Fig. 5a,f,g,h). This result highlights the significance of cellular content and corroborates findings in a previous ex vivo DTI study on cell migration in brain tumours^30^, where the authors saw a decrease in FA and increase in MD when cells migrated out of a region of interest.

Vascular SMCs on average are 200 μm long and 5 μm in diameter^31^, while a single collagen fibril diameter is 80 ± 11 nm and a fibre bundle is approximately 5.1 ± 6.1 μm^32^. SMCs are responsible for the turnover of the extracellular matrix, including collagen, and therefore their orientation aligns with that of collagen. Together these components form the helical matrix of arterial tissue which has been well documented^5,6^. Using the 3D root-mean square equation^33^ ($r=\sqrt{6D\Delta }$), the gradient interval time used in this study (Δ = 8.802 ms) and the measured MD in PBS, a diffusing water molecule could travel approximately 9.8 μm, suggesting sensitivity to diffusion at the scale of both SMCs and collagen.

In the absence of obstructing boundaries, protons diffuse freely in all directions. The presence of boundaries, such as SMCs or collagen fibres, cause either restricted or hindered diffusion. Intracellular diffusion is typically regarded as restricted diffusion as the molecules are trapped within the cell membrane and have limited space to diffuse within^34^. Hindered diffusion arises when the diffusion of water molecules is impeded by boundaries, such as collagen fibres, SMCs or elastin fibres, which reduce their net displacement. Generally, extracellular diffusion in biological tissue is characterised as hindered diffusion^34,35^. The cell membrane is composed of a phospholipid bilayer which is selectively permeable and, due to the polar nature of water molecules, limits the exchange rate of molecules across the membrane from intra- to extracellular space and vice versa^36^. In contrast, aquaporins are channel proteins that act as transmembrane water channels and their gating (open or closed) is a result of biochemical signalling. It has been shown in both rat and human vascular SMCs that aquaporin-1 plays an important role in vascular function—specifically in development and injury^37^. Within the scale of diffusion presented here, we see the combined effects of both intra- and extracellular diffusion^38^ and changes in their profile as components are removed. It should be noted that the exact effect the enzymatic treatments have on the integrity of the cell membrane—for example on aquaporin gating—is unknown and requires further research. There has also been significant research investigating the choice and use of higher order DTI models to specifically characterise different diffusion compartments, which may provide further insight into the microstructural changes seen in the arterial models presented in this study^39–41^.

The removal of SMCs from arterial tissue resulted in a drastic decrease in FA, as can be seen in the decellularised tissue model (Fig. 3). The high FA measured in the collagen degraded vessels suggests that SMCs are the main contributors to the overall anisotropic diffusion. Both the intra- and extracellular hindered diffusion associated with their presence have a greater impact on anisotropic diffusion than hinderance from interactions with collagen fibres alone. Removal of elastin from the artery resulted in the most isotropic diffusion of all models, seen by low FA and the highest MD. While the quantity of cells did not change in the elastin degraded model, as previously mentioned, the removal of elastin resulted in drastically increased extracellular space and decreased cell density—this can be seen histologically by H&E (Fig. 1a,c). SMCs attach to the concentric elastin lamellae and are embedded between collagen fibres^11^. The removal of elastin disrupted this relationship and with the drastically increased extracellular space and lower cell density, proton mobility increased and, therefore, increased the MD^42^. This structural change can be seen plainly in the FA (Fig. 3d) and MD (Fig. 4d) maps and is confirmed histologically (Fig. 1c). The MD of the elastin degraded model is higher than that of the decellularised model suggesting that not only does the less densely packed cell content affect the DTI metrics compared to native, but the decrease in hindered diffusion from the removed elastin molecules also plays a significant role in this isotropic response.

It is worth noting that all the tissue models displayed a decrease in GAG content (Fig. 1, bottom row). A study by Bartholomew and Anderson^43^ demonstrated that proteoglycans, the proteins GAGs attach to, coat collagen type III in the media, which in turn coats the elastin fibres. This suggests it is not possible to avoid the disruption and depletion of GAGs in arterial tissue when removing collagen or elastin. In cartilage, Xia et al.^42^ illustrated that the MD has no direct correlation to GAG content but instead they proposed that the space left from degraded and removed macromolecules allows for increased diffusivity—which has since been demonstrated^44^. To the author’s knowledge, no studies exist examining the influence that GAGs have on diffusion in arterial tissue; however, while proteoglycans show no preferred orientation and therefore shouldn’t influence FA, as previously suggested, their removal could increase MD as a result of the open extracellular space left behind upon their removal.

The main constituents of native PCaA are SMCs, elastin and collagen. When fixing native arterial tissue, these constituents were unaffected and there was no effect on the FA, MD or tractography. While multiple studies have reported increased MD in cardiac tissue when fixed^9,45,46^, the length of fixation, concentration of fixative and time after fixation in cardiac tissue have resulted in considerable variation in measured FA and MD^47,48^. One study on fixed tissue observed an initial decrease in the MD followed by an increase after 15 days^47^, whilst it has also been shown that increased exposure to fixatives can cause cell membrane degradation by the depletion of lipid membranes through carbon double bond reactions^49^. The effect of fixation on sample dimensions has been well documented, with tissue shrinkage a known side effect^50,51^. Our results showed no significant difference between fresh and fixed tissue with respect to the FA, MD and tractography. The length of our fixation protocol, seven days, is likely short enough to avoid any membrane degradation and therefore had no significant effect on diffusivity. This time frame has also been shown to minimise the effect of shrinkage from fixation, however different concentrations of formalin should be investigated in the future^51^.

Previous studies in arterial tissue have looked at the structure of native arterial tissue as well as how storage and preparation for imaging affect the diffusion profiles. While the fibre angles have not been quantified in the present study, the helically aligned tractography of native and collagen degraded tissue corroborate previous studies highlighting the helical arrangement of SMCs and collagen^5,6^. The results from the tractography analysis provide visualisation of the significance of cell content on the diffusion profile. It is demonstrated in this study that, within arterial tissue, tractography is sensitive to cellular orientation, more so than just collagen fibre arrangement (Fig. 5a,c,e). Histological analysis demonstrates that SMCs and collagen follow the same circumferential arrangement in native arterial tissue (Fig. 6a) and tractography yields good visualisation of that structure. However, for the collagen degraded vessels (Fig. 6d) the tractography is a representation of cellular alignment alone.

While all samples in this study were porcine carotids, the study did not control for the differences between proximal or distal sections of these carotids. Carotids are elastic arteries; however, more distal sections can be more muscular, thus containing less elastin in the medial layer^10^. While this can explain visual differences between arteries in Figs. 3 and 4 (c and e), the treatments performed were designed and confirmed to selectively remove all of a specific microstructural component from a given vessel.

While a 2D slice selective scan would certainly be faster, crosstalk between slices can occur and may require slice gaps which are not ideal for investigating tractography. Additionally, while EPI acquisitions offer faster scan times, they can also introduce further distortions and artefacts. With these variables in mind, the 3D spin echo DTI sequence was used in this study as it provided high resolution images with minimal distortion or artefacts and good SNR. However, the lengthy duration of the scan posed multiple limitations. It would be expected to have some tissue degradation during the scan time; however, all samples were treated the same and imaged for the same scan time and there was no evidence of degradation histologically. Additionally, a limited number of diffusion directions and unweighted images were acquired due to the long scan duration. While the same number of diffusion directions and unweighted diffusion images has been used previously in arterial tissue and shown microstructurally accurate results^6,22^, it should be noted that the ratio of unweighted to weighted diffusion scans is not optimal in this study^52^. Ranges of FA and MD from a previous study on arterial tissue using multiple b-values and up to 128 b-directions agree well with the measurements made in this study^8^. While healthy arterial tissue microstructure is quite homogeneous and crossing fibres would not be common, the low number of diffusion directions can bias DTI derived metrics and should be investigated further to prevent potential bias in diseased arterial tissue where the homogeneity of microstructure is disrupted^53^.

Microstructural changes in arterial tissue can have significant implications for the mechanical functionality of the tissue^54^, as well as often being a precursor to disease progression^4^. On this basis and considering the characterisation of arterial microstructure presented in this study, DTI has the potential to provide unique biomarkers for the integrity of arterial tissue. Atherosclerosis is a chronic immunoinflammatory, fibroproliferative disease which starts with the adhesion of low-density lipoproteins to the intimal layer of the arterial wall^3^. After this initial step, macrophages and foams cells rapidly accumulate at the intimal layer and migrate into the intimal-medial layer boundary resulting in a continually changing microstructure as disease progresses. Morphologically, the first signs of an atherosclerotic plaque are the thickening of the intima, followed by the well-known formation of a lipid core and fibrous cap^55,56^. Microstructurally, these different regions have altered quantities and arrangements of SMCs, collagen and elastin. The thickened intima typically shows a decrease in SMC content^55^, the lipid core highlights the displacement of SMCs by foam cells^56^, and the fibrous cap, which covers the lipid core^57^, has been shown to have quite variable SMC content depending on location^58^. The demonstrated sensitivity of DTI to SMC content in arterial tissue in this study suggests that it may be an ideal metric to identify such early indicators of disease driven microstructural changes. Additionally, other cardiovascular diseases—such as aneurysms—have shown significant fragmentation of the elastic lamellae which can cause catastrophic failure of the arterial wall^59^. Changes in the key microstructural components of arterial vessels can lead to significant mechanical failings^54^ and identifying these changes using imaging biomarkers, offers potential insight into the mechanical integrity of the arterial wall in atherosclerotic^13,60,61^ and/or aneurysmal^59^ tissue.

Few studies have looked at the implementation of diffusion imaging in vivo for carotid artery and atherosclerotic plaque visualisation^62–65^ as there are many elements which make clinical translation challenging. The high resolution, lack of physiological motion^66^ and extended scan time in this study allowed for a detailed look at the vessel microstructure, which would be necessary for investigating regions of atherosclerotic plaques. Despite the idealised ex vivo experimental set up, the work presented here highlights the promise for DTI metrics to yield valuable insight into arterial microstructure which could ultimately provide novel insight into diseased tissue morphologies. For example, recent in vivo studies have used quantitative susceptibility mapping to investigate gross morphological features^67–69^ and inflammation^70^ in atherosclerotic plaques, but this approach also has potential to provide markers of tissue microstructure and integrity^21^. Ideally, a combination of methods which allow for the full characterisation of the microstructure within the vessel wall would provide the insight needed to better inform the risk of atherosclerotic plaque rupture. This study establishes the influence of key microstructural components on diffusion metrics in arterial tissue and highlights the potential of DTI for identifying disease driven changes in arterial microstructure.

## Methods

All methods were carried out in accordance with relevant guidelines but as the animal tissue used in these experiments was obtained from a licensed slaughterhouse, additional approval for use by a licensing committee was not required.

### Specimen preparation

**Figure 7:**
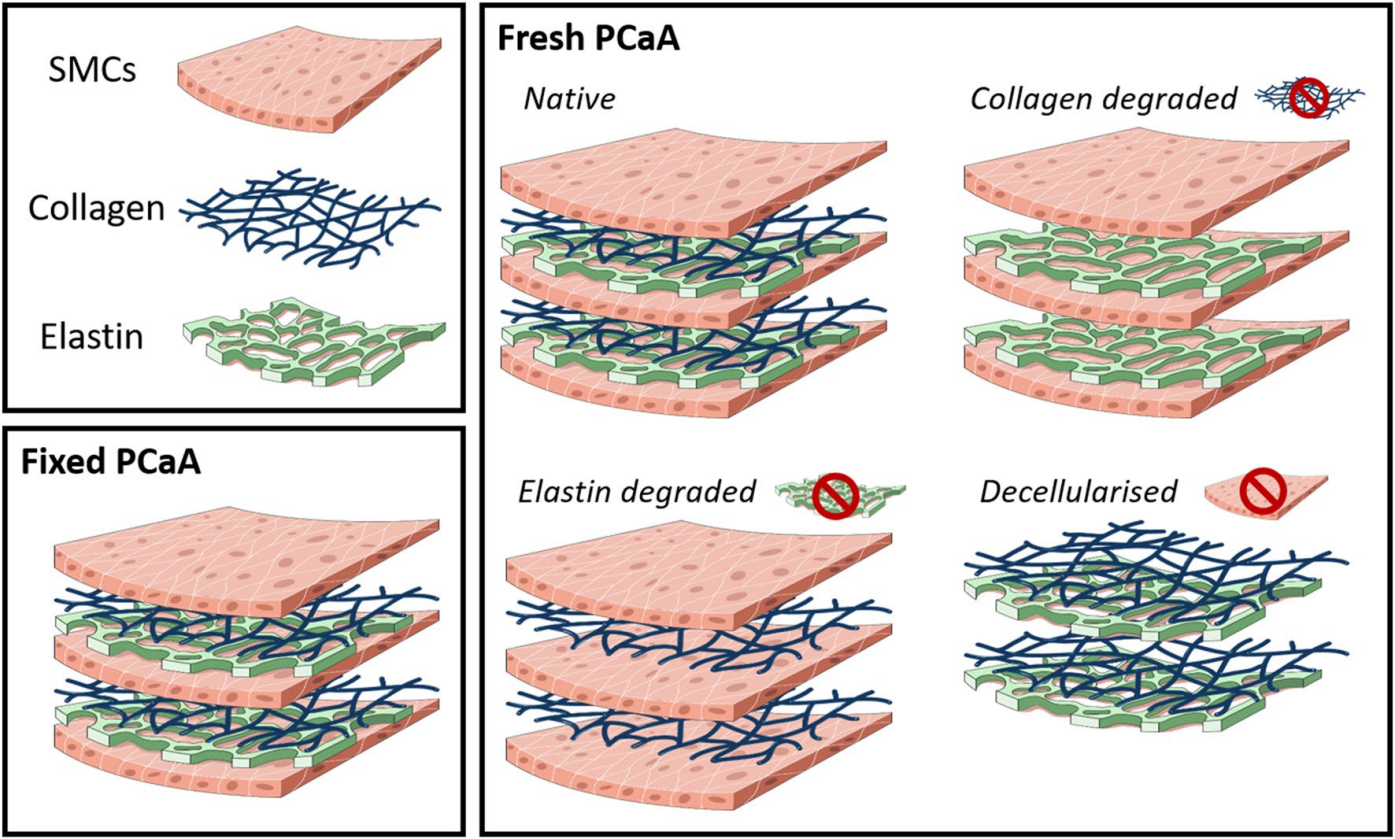
Schematic of the arterial tissue models used in this study. Four models were made from fresh tissue and had the selective removal of components—native, collagen degraded, elastin degraded and decellularised porcine carotid artery. Fixed native porcine carotid artery was also investigated.

PCaA of 6-month-old healthy Large White pigs all from the same abattoir were excised and within 3 h of sacrifice all arteries were cleaned of connective tissue and cryopreserved together at a controlled rate of − 1 ºC/min to − 80 ºC in tissue freezing media. Tissue freezing medium was made up of 500 mL Gibco RPMI 1640 Medium (21875034, BioSciences), 19.6 g sucrose (S0389, Sigma) and 73.3 mL of the cryoprotectant dimethylsulfoxide (PIER20688, VWR International). Cryoprotectants have been shown to prevent the formation of ice crystals and therefore maintain tissue microstructure during freezing^71,72^. Upon thawing at 37 ºC, vessels were rinsed in PBS to remove any excess cryoprotectant. All vessels were cryopreserved prior to treatment and imaged directly after treatment to ensure consistent preparation. Five tissue models were used in this study (n = 6 for each): native, fixed native, collagen degraded, elastin degraded and decellularised PCaA. Native, decellularised, collagen degraded and elastin degraded PCaA were imaged fresh (unfixed) while the fixed native was imaged after formalin fixation. Table 1 and Fig. 7 outline these models and their respective treatments. Upon thawing and after tissue treatments all samples underwent five PBS rinses in order to ensure any excess reagents were removed prior to being placed in fresh PBS for imaging.

**Table 1:** Five different PCaA tissue models and the respective treatments.

PCaA tissue model	Treatment
Native	N/A
Fixed native	4% formalin (HT501128, Sigma) fixation for 7 days at 4 ºC
Collagen degraded	1000 U/ml purified collagenase (CLSPA, Worthington Biochemical Corporation) in MgCl_2_ + CaCl_2_ supplemented PBS (D8662, Sigma) at 37 ºC for 28 h on a rotator
Elastin degraded	10 U/ml purified elastase (ESFF, Worthington Biochemical Corporation) with 0.35 mg/ml trypsin inhibitor (10109886001, Sigma) in Dulbecco’s Modified Eagle Medium, high glucose, GlutaMAX (61965026, BioSciences) at 37 ºC for 3.5 h
Decellularised	0.1 M sodium hydroxide (S8045, Sigma) perfused through native vessels via a peristaltic pump at 2 Hz for 15 h, followed by 0.1 M sodium chloride (S3014, Sigma) for 32 h—all with a pressure of 100 mmHg during perfusion; then treated with 10 µl/ml DNAase (LS006343, Worthington Biochemical Corporation) and 2 µl/ml primicin (Ant-pm-2, InvivoGen) at 37 ºC for 19 h^72^

### MR data acquisition

A small bore (35 cm) horizontal 7 T Bruker BioSpec 70/30 USR system (Bruker, Ettlingen Germany) equipped with a receive only 8-channel surface array coil, birdcage design transmit coil, shielded gradients (maximum strength 770 mT/m) and Paravision 6 software was used for all imaging. All vessels were positioned using a custom-made 3D printed holder placed in a 50-ml falcon tube and immersed in fresh PBS prior to imaging at room temperature. A conventional 3D spin-echo DTI sequence with monopolar gradients was used with the following parameters: TE/TR: 17.682/1000 ms, image size: 96 × 96 × 60, field of view: 30 × 30 × 18.75 mm^3^, isotropic resolution: 312.5 × 312.5 × 312.5 μm^3^. One b-value of 0 s/mm^2^ was acquired, with a b-value^73,74^ of 800 s/mm^25^ subsequently applied in 10 isotopically distributed directions^53^. Standard values for this sequence were used for the diffusion gradient separation, Δ (8.802 ms), and the diffusion gradient duration, δ (3.8 ms), and the total acquisition time was 17 h and 36 min.

### Image reconstruction and processing

All raw data was denoised^75^ and corrected for Gibbs ringing^76^ in MRtrix3^77^ (http://www.mrtrix3.org) prior to the mono-exponential tensor model^73,74^ fitting in ExploreDTI^78^. The mono-exponential equation expands to incorporate the diffusion tensor and b-matrix—which characterises the diffusion sensitivity from the effects of the diffusion gradients, imaging gradients and cross-terms^74^. From the tensor, the MD and FA were calculated in ExploreDTI. The MD represents the total diffusion within a voxel, while FA is indicative of the degree of anisotropic diffusion occurring within a voxel on a scale of 0–1^79,80^.

### Regional analysis and tractography

Regions of interest were manually drawn within the media of each vessel using an image created from the mean of the b = 800 s/mm^2^ images. Mean values of FA and MD were calculated from multiple slices within these regions for each vessel. Tractography was similarly performed for all vessels (n = 6 per tissue model group) within ExploreDTI and all parameters used are presented with the corresponding results. Representative vessels are presented for each group alongside FEFA maps and quantitative tractography metrics were calculated for all vessels.

### Histology

For histological processing, all tissue model samples were fixed in 4% formalin for seven days at 4 ºC prior to stepwise dehydration in ethanol to xylene. Once dehydrated, all samples were embedded in paraffin wax and sectioned at 8 µm thick slices prior to staining. All stains, their purpose and required imaging are listed in Table 2*.* All imaging was done using an Olympus BX41 microscope with Ocular V2.0 software. PLM uses a polarised filter and two images 90º to each other to maximise the birefringence of collagen for visualisation. Representative histological images are presented.

**Table 2:** Histological stains used in this study, their visualisation and how they are imaged.

Stain	Visualisation	Imaging
Alcian blue	Sulphated mucans, such as glycosaminoglycans (GAGs), stain blue	Brightfield
Haematoxylin and eosin (H&E)	Haematoxylin stains acidic structures, like cell nuclei, purple-blue and eosin stains basic features, like cytoplasmic filaments, membranes and fibres, pink	Brightfield
Picrosirius red	Collagen stains red, while PLM selectively highlights collagen birefringence allowing for visualisation of the orientation	Brightfield + PLM^81^
Verhoeff’s	Elastin stains black	Brightfield

### Statistical analysis

Statistical analysis was performed with GraphPad Prism (Version 8). One-way ANOVA with Tukey’s post hoc test for multiple comparisons was used to analyse the variance between groups and determine significance. All numerical and graphical significance is shown as mean ± standard deviation, n = 6 within each tissue model group and α = 0.05 for all tests.

## Data Availability

The data (DTI-scans) for each tissue model in the current study are available from the corresponding author on reasonable request.
